# Efficacy and safety of tocilizumab in european children with systemic onset juvenile idiopathic arthritis

**DOI:** 10.1186/1546-0096-9-S1-P135

**Published:** 2011-09-14

**Authors:** O Nemiche, R Dagner, P Quartier, R Cimaz, O Richer, P Pillet, M Hofer

**Affiliations:** 1 Department of Immuno-Hémato-Rhumatology, Necker Enfants Malades Hospital, Paris France; 2 Service of Pediatric Rheumatology, Florence Italy; 3 Service of Pediatric Rheumatology, CHU Bordeaux, France; 4 Service of Pediatric Rheumatology, CHU, Vaudois, Lausanne, Geneva, Swiss

## Background

The anti- IL-6 Receptor monoclonal antibody Tocilizumab (TCZ) has demonstrated its efficacy in Japanese children with systemic onset juvenile idiopathic arthritis (SJIA).

## Aim

To evaluate the efficacy and safety of TCZ in European patients with active SJIA outside a clinical trial (off-label use).

## Patients and methods

Retrospective review of the files of the patients treated by TCZ in two French, one Swiss and one Italian center. Response to treatment was defined as control of the fever and systemic features for at least 7 days, Improvement of the pediatric ACR score and normalization of erythrocyte sedimentation rate (ESR) and C-reactive protein (CRP) were also considered.

## Results

18 patients aged 4-15 years were included. The median disease duration at treatment onset was 4.7 years (0.4-8.8). The median dose of prednisone at TCZ onset was 0.63mg/kg/d. Four patients were also on MTX. Fourteen patients had previously failed to respond one or several biologics, including anti-TNF alpha in 10 cases, anakinra in 14 and canakinumab, in 4. All patients but 4 had active systemic and polyarticular features at TCZ onset. The doses of TCZ ranged between 6 and 12 mg/kg every other week at treatment onset. The mean follow-up on TCZ was 18.2 months (range0.5-48). 90% improvement of the pediatric ACR score was achieved by 9 patients after 3 months. The dose of steroids was tapered in most cases (11/14) within 3 months, and five patients could discontinue steroid treatment after 12 months. TCZ treatment was withdrawn in 5 children for adverse events: anaphylactic reaction in 3 cases, skin vasculitis in 2 other cases.

## Conclusion

In this study TCZ treatment was effective in most patients with SJIA who previously failed one or several biologics. Adverse events require particular attention since almost one third of patients had to discontinue treatment.

